# Supplementary material for: Iterative improvement in the automatic modular design of robot swarms
Source: PeerJ Comput Sci. 2020 Dec 7;6:e322. doi: 10.7717/peerj-cs.322 (PMC7924708; doi:10.7717/peerj-cs.322)
Supplement: Supplemental Information 3 [file peerj-cs-06-322-s003.zip › argos3/doc/api/standalone/a00515_source.html]

ARGoS: plugins/robots/generic/control\_interface/ci\_range\_and\_bearing\_actuator.h Source File


- Main Page
- Related Pages
- Namespaces
- Classes
- Files

- File List
- File Members

# plugins/robots/generic/control\_interface/ci\_range\_and\_bearing\_actuator.h

Go to the documentation of this file.

```
00001 
00007 #ifndef CI_RANGE_AND_BEARING_ACTUATOR_H
00008 #define CI_RANGE_AND_BEARING_ACTUATOR_H
00009 
00010 namespace argos {
00011    class CCI_RangeAndBearingActuator;
00012 }
00013 
00014 #include <argos3/core/control_interface/ci_actuator.h>
00015 #include <argos3/core/utility/datatypes/byte_array.h>
00016 
00017 namespace argos {
00018 
00019    class CCI_RangeAndBearingActuator : public CCI_Actuator {
00020 
00021    public:
00022 
00023       virtual ~CCI_RangeAndBearingActuator() {}
00024 
00025       size_t GetSize() const;
00026 
00027       void SetData(const CByteArray& c_data);
00028 
00029       void SetData(size_t un_idx,
00030                    UInt8 un_value);
00031 
00032       void ClearData();
00033 
00034 #ifdef ARGOS_WITH_LUA
00035       virtual void CreateLuaState(lua_State* pt_lua_state);
00036 #endif
00037 
00038    protected:
00039 
00040       CByteArray m_cData;
00041 
00042    };
00043 
00044 }
00045 
00046 #endif
```

---

Generated on 10 Jul 2018 for ARGoS by 
 1.6.1
